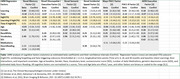# Remote Computerized Cognitive Test Battery Reflects Subjective Cognitive Complaint

**DOI:** 10.1002/alz70857_105754

**Published:** 2025-12-26

**Authors:** Timothy J Herron, Jas M. Chok, Sandy J. Lwi, Maria G Spinelli, Krista Schendel, Brian Curran, Juliana Baldo

**Affiliations:** ^1^ Veterans Affairs Northern California Health Care System, Martinez, CA, USA; ^2^ Palo Alto University, Palo Alto, CA, USA; ^3^ University of California, Davis, CA, USA

## Abstract

**Background:**

Recent advances in the treatment of dementia have accelerated the need for sensitive markers of very early cognitive decline. Here, we look at the utility of primary and secondary cognitive test metrics obtained using a remotely proctored, computerized test battery in a longitudinal study of cognitively healthy older participants. Specifically, we are looking for cognitive markers related to subjective cognitive complaints.

**Method:**

We assessed 309 cognitively healthy older participants from Northern California (62‐89 y.o., 113 F, edu 15.2 yr, 220 white) using the California Cognitive Assessment Battery (CCAB) at five timepoints across 30 months. Three traditional aggregate factors were created from CCAB test scores as defined in previous publications. Four novel cognitive factors were determined in a previous study by exploratory factor analysis on a separate cohort of 90 subjects (30‐62 y.o.), and include selected timing and other secondary metrics that are not components of the traditional aggregate factors. Cognitive factor scores were compared to subjective memory complaints defined using the Cognitive Failures Questionnaire (CFQ). We used linear mixed model (LMM ; *lme4* in R) analyses and latent class mixed models (LCMM ; *lcmm* & *flexmix* in R) to analyze this longitudinal dataset.

**Result:**

LMM analyses showed that the novel Memory, Visuospatial/Executive, and Incidental Speed factors, plus a traditional PACC‐R factor were significantly correlated with elevated CFQ subscores (see Table). No factor showed significant interactions of elevated CFQ with either test learning (based on a baseline two‐day test‐retest session) or in factor declines across 30 months. The LCMM analyses showed mixed results, with the *lcmm* analysis producing no reliable latent models that separate participants with elevated CFQ scores from those without. However, the *flexmix* analysis reliably classified subjects into 2 groups primarily separated by vocabulary, age, and race; but with group membership only weakly correlating with elevated CFQ.

**Conclusion:**

Our findings showed that novel cognitive factors defined using CCAB were related to participants’ subjective complaints, such as forgetfulness. Such cognitive markers could potentially be used as partial early markers of cognitive decline. Work is ongoing to validate the use of these novel metrics in larger, more diverse populations and within clinical (MCI) populations.